# Supplementary material for: Heat stress does not induce wasting symptoms in the giant California sea cucumber (Apostichopus californicus)
Source: PeerJ. 2023 Feb 7;11:e14548. doi: 10.7717/peerj.14548 (PMC9912942; doi:10.7717/peerj.14548)
Supplement: Supplemental Information 1 [file peerj-11-14548-s001.docx]

**Supplementary Information**

Table S1. Results from logistic regression model examining which variables best predicted sea cucumber mortality

| Variable | Coefficient | Std. Error | T | p |
| --- | --- | --- | --- | --- |
| (intercept) | -21.146 | 170.49 | -0.124 | 0.902 |
| Treatment: 17C | 0.0363 | 246.59 | 0 | 0.999 |
| Treatment: 22C | 14.72 | 170.42 | 0.086 | 0.932 |
| Weight | 0.0103 | 0.00553 | -1.86 | 0.0678 |

*p < 0.05, **p < 0.01

**Table S2**. Results of ordinal regression model examining the effect of treatment and experiment day on sea cucumber stiffness.

| Variable | Coefficient | Std. Error | T | p |
| --- | --- | --- | --- | --- |
| Treatment: 17C | -2.99 | 0.575 | -5.20 | 1.99e-07 ** |
| Treatment: 22C | -4.05 | 0.606 | -6.68 | 2.44e-11 ** |
| Day 2 | -0.976 | 0.524 | -1.86 | 0.0627 |
| Day 3 | -2.30 | 0.528 | -4.35 | 1.37e-05 ** |
| Day 4 | -2.22 | 0.527 | -4.21 | 2.50e-05 ** |
| Day 5 | -2.12 | 0.541 | -3.93 | 8.66e-05 ** |

*p < 0.05, **p < 0.01

**Table S3**. Results from a backwards-selected logistic regression model examining the effect of evisceration as a function of defecation status and weight. Asterisks indicate significant effects.

|  | Coefficient | Std. Error | t-value | p |
| --- | --- | --- | --- | --- |
| (Intercept) | 1.713 | 1.128 | 1.52 | 0.135 |
| Defecating (yes) | -2.78 | 1.12 | -2.48 | 0.0163 * |
| Weight | -0.00431 | 0.00203 | -2.12 | 0.0383 * |

*p < 0.05, **p < 0.01


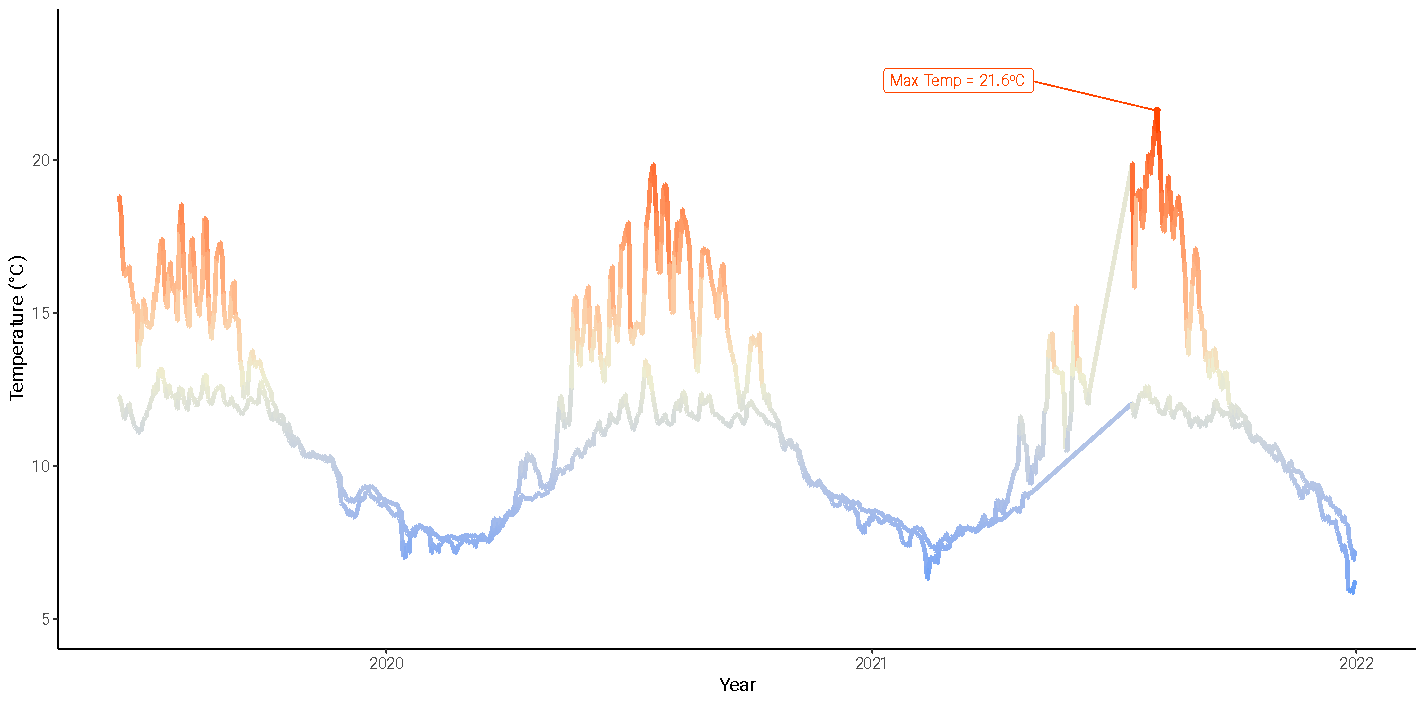


Figure S1. Average seasonal temperatures at 5m depth (top line; device ID: 24124) and 20m depth (bottom line; device ID: 24001) between 2019-2022 in the Strait of Georgia where sea cucumber wasting was observed after a maximum temperature of 21.6ºC was reached on August 04, 2021. Line colour represents deviation from average temperature (grey), indicating the magnitude of warming (red) or cooling (blue) of the sea. Data obtained from Ocean Networks Canada.

Figure S2. Average treatment temperatures (black lines) in control treatment (Grey, 12ºC) warm (red; 17ºC), or heat wave (orange; 22ºC). Temperatures were gradually increased over a 24-hour period in the sea table water baths (day 1). The water remained at target temperatures for 79 hours, after which they were lowered back to the control temperature of 12ºC over 9-hours. Days 5 through 12 were a recovery period where all sea cucumber holding containers were maintained on the flow through system..
